# LTP cross-reactivity - primary sensitization to mugwort pollen LTP Art v 3, facilitates subsequent sensitisation to peach LTP Pru p 3 in mice

**DOI:** 10.1186/2045-7022-4-S2-O14

**Published:** 2014-03-17

**Authors:** Andrea Wangorsch, Stefan Schülke, Gabriele Gadermaier, Melanie Albrecht, Michael Wallner, Stefanie Randow, Sonja Wolfheimer, Jonas Lidholm, Iris Lauer, Fatima Ferreira, Masako Toda, Stefan Vieths, Gerald Reese, Stephan Scheurer

**Affiliations:** 1 Paul-Ehrlich-Institut, Division of Allergology, Langen, Germany; 2 University of Salzburg, Department of Molecular Biology, Salzburg, Austria; 3 Thermo Fisher Scientific, Research and Development, Uppsala, Sweden; 4 Paul-Ehrlich-Institut, Junior Research Group 1 “Experimental Allergology”, Langen, Germany

## 

Non-specific lipid transfer proteins (nsLTPs) are important pollen and food allergens in the Mediterranean area. Peach LTP Pru p 3 is considered as primary sensitizer inducing LTP cross-reactive IgE antibodies. Despite the evidence on the importance of Pru p 3, it is unclear whether reactivity to multiple LTPs is due to antibody cross-reactivity or to independent sensitization events. Aim of the study was (1) to compare antigenicity of LTPs from pollen and food, and (2) to investigate whether primary sensitization to LTPs promotes secondary sensitization to LTPs from unrelated sources. IgE sensitization to peach LTP was compared in different mouse strains (C3H/HeJ, BALB/c, CBA/J). IgE high responder mice (CBA/J) were used to compare the antigenicity and IgE cross-reactivity of LTPs from pollen (mugwort/Art v 3, plane tree/Pla a 3, ragweed/Amb a 6) and foods (peach/Pru p 3, cherry/Pru av 3, hazelnut/Cor a 8). To investigate whether an established Th2 immune response to LTPs facilitates a secondary immune response and cross-reactivity to unrelated LTPs, CBA/J mice were immunized with (1) Pru p 3 followed by Art v 3, (2) Art v 3 followed by Pru p 3, and (3) a mixture of Pru p 3 and Art v 3. Induction of Pru p 3 and Art v 3 specific IgE and IgG titers, and cross-reactivity between the two LTPs were determined by ELISA. CBA and C3H mice showed strong IgE-responses to Pru p 3, whereas BALB/c mice did not. Remarkably, a species-specific immune response lacking any IgE- and IgG-cross-reactivity was observed for all tested LTPs except for Pru p 3 and Pru av 3. Although LTPs show high structural similarity, Pru p 3 and Art v 3 displayed different antigenicity. In comparison to Art v 3, Pru p 3 showed much lower IgE-sensitizing capacity. In contrast, IgE response to Pru p 3 was promoted in mice previously sensitized to Art v 3, which was even the case upon concurrent administration of both LTPs. Neither sequential immunization nor immunization with the mixture induced IgE antibodies to unrelated LTPs, e.g. Cor a 8. In summary, the immune response to LTPs in mice varied strongly between strains. The LTPs studied, displayed different immunogenic properties and did not induce cross-reactive IgE antibodies. Primary sensitization to the pollen LTP Art v 3 conditioned the animals for subsequent sensitization to the food LTP Pru p 3. The extent to which the findings are applicable to the manifestation of clinical cross-reactivity to LTPs in humans needs to be investigated.

